# Cladieunicellins M–Q, New Eunicellins from *Cladiella* sp.

**DOI:** 10.3390/md12042144

**Published:** 2014-04-08

**Authors:** Tsung-Hung Chen, Wu-Fu Chen, Zhi-Hong Wen, Mei-Chin Lu, Wei-Hsien Wang, Jan-Jung Li, Yang-Chang Wu, Ping-Jyun Sung

**Affiliations:** 1 Graduate Institute of Marine Biotechnology, Department of Life Science and Institute of Biotechnology, National Dong Hwa University, Pingtung 944, Taiwan; E-Mails: a610162002@gmail.com (T.-H.C.); jinx6609@nmmba.gov.tw (M.-C.L.); 2 National Museum of Marine Biology and Aquarium, Pingtung 944, Taiwan; E-Mails: whw@nmmba.gov.tw (W.-H.W.); jj@nmmba.gov.tw (J.-J.L.); 3 Department of Neurosurgery, Kaohsiung Chang Gung Memorial Hospital and Chang Gung University College of Medicine, Kaohsiung 833, Taiwan; E-Mail: ma4949@adm.cgmh.org.tw; 4 Department of Marine Biotechnology and Resources, Asia-Pacific Ocean Research Center, National Sun Yat-sen University, Kaohsiung 804, Taiwan; E-Mail: wzh@mail.nsysu.edu.tw; 5 School of Pharmacy, College of Pharmacy, China Medical University, Taichung 404, Taiwan; 6 Chinese Medicine Research and Development Center, China Medical University Hospital, Taichung 404, Taiwan; 7 Center for Molecular Medicine, China Medical University Hospital, Taichung 404, Taiwan; 8 Graduate Institute of Natural Products, Kaohsiung Medical University, Kaohsiung 807, Taiwan

**Keywords:** eunicellin, *Cladiella*, cladieunicellin, cytotoxicity

## Abstract

Five new 7α-hydroxyeunicellin-based diterpenoids, designated as cladieunicellins M–Q (**1**–**5**), were isolated from a Formosan octocoral *Cladiella* sp. The structures of **1**–**5** were elucidated on the basis of spectroscopic methods and by comparison of the data with those of the related metabolites. Cytotoxicity of metabolites **1**–**5** against the human leukemia Molt 4 and HL 60 is also described. Among them, compounds **1**, **3** and **5** exhibited moderate cytotoxicity toward Molt 4 cells with IC_50_ values 16.43, 14.17 and 15.55 μM, respectively. Preliminary SAR (structure activity relationship) information was obtained from these compounds and their analogues.

## 1. Introduction

**Chart 1:**
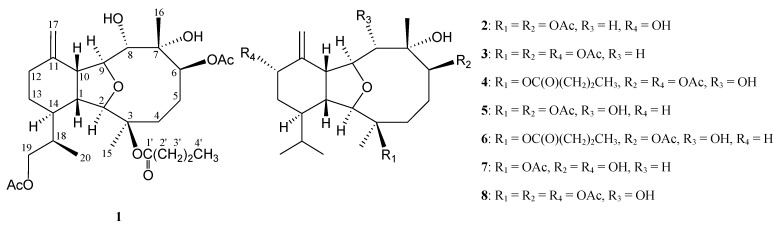
The structures of cladieunicellins M–Q (**1**–**5**), krempfielins C and L (**6** and **7**) and cladieunicellin L (**8**).

During the course of our search for novel metabolites from marine invertebrates of Taiwanese waters, a series of eunicellin-type diterpenoids including cladieunicellins A–J, have been isolated from a soft coral identified as *Cladiella* sp. (family Alcyoniidae) collected in Taiwan waters [1,2,3]. Because of our interest in the chemistry of new natural products, the continuing investigation on the chemical constituents of the soft coral *Cladiella* sp. was carried out and resulted in the isolation of five new eunicellin-based diterpenoids, cladieunicellins M–Q (**1**–**5**) (Chart 1). This paper deals with the isolation, structure elucidation and cytotoxicity of compounds **1**–**5**.

## 2. Results and Discussion

Cladieunicellin M (**1**) was obtained as colorless oil and its molecular formula of **1** was established as C_28_H_44_O_9_ (7° of unsaturation) by the HRESIMS at *m/z* 547.28760 (calcd for C_28_H_44_O_9_Na, 547.28775). The IR absorptions at υ_max_ 3462 (broad) and 1734 cm^−1^ revealed the presence of hydroxy and ester carbonyl functionalities. The ^13^C NMR of **1** showed 28 carbon signals (Table 1), which were assigned with the assistance of the DEPT spectrum to six methyls, seven sp^3^ methylenes (including an oxymethylene), an sp^2^ methylene, eight sp^3^ methines (including four oxymethines), two sp^3^ oxygenated quaternary carbons and four sp^2^ quaternary carbons (including three carbonyls). The ^13^C resonances at δ_C_ 172.3, 171.9 and 171.2 demonstrated the presence of three ester carbonyls. Two of these signals were identified as acetate carbonyls by the presence of two methyl resonances in the ^1^H NMR spectrum at δ_H_ 2.09 and 2.08 (each 3H × s) and the other one was identified as an *n*-butyrate carbonyl by the presence of seven contiguous protons at δ_H_ 0.99 (3H, t, *J* = 7.2 Hz), 1.66 (2H, m) and 2.32 (2H, m). From the ^13^C NMR data, an exocyclic carbon-carbon double bond was deduced from the signals at δ_C_ 147.8 (C-11) and 111.1 (CH_2_-17), and confirmed by two olefin proton signals at δ_H_ 4.91 (1H, br s, H-17) and 4.79 (1H, dd, *J* = 2.0, 1.6 Hz, H-17) in the ^1^H NMR spectrum. In addition, a suite of resonances of proton signals at δ_H_ 3.84 (1H, dd, *J* = 8.8, 6.8 Hz, H-9), 3.57 (1H, s, H-2), 3.38 (1H, dd, *J* = 7.2, 6.8 Hz, H-10) and 2.23 (1H, dd, *J* = 10.8, 7.2 Hz, H-1) and carbon signals at δ_C_ 92.7 (CH-2), 81.5 (CH-9), 53.5 (CH-10) and 45.1 (CH-1), indicated the presence of a tetrahydrofuran moiety. Comparison of the ^13^C NMR and DEPT spectra with the molecular formula indicated that there must be two exchangeable protons, requiring the presence of two hydroxy groups. From the above data, compound **1** was proven to be a diterpenoid with three rings.

**Table 1:** ^1^H (400 MHz, CDCl_3_) and ^13^C (100 MHz, CDCl_3_) NMR data, ^1^H–^1^H COSY and HMBC correlations for eunicellin **1**.

Position	δ_H_ ( *J* in Hz)	δ_C_, Multiple	^1^H–^1^H COSY	HMBC
1	2.23 dd (10.8, 7.2)	45.1, CH	H-10, H-14	C-3, -9, -10, -14, -18
2	3.57 s	92.7, CH	n.o. *^a^*	C-1, -3, -10, -14, -15
3		86.0, C		
4	2.59 dd (13.6, 7.2)	35.4, CH_2_	H_2_-5	C-2, -3, -6, -15
	2.00 m			
5	1.55–1.40 m	28.5, CH_2_	H_2_-4, H-6	C-3, -6, -7
6	5.72 d (4.8)	82.2, CH	H_2_-5	C-4, -5, -7, -16, acetate carbonyl
7		78.3, C		
8	3.58 dd (9.6, 8.8)	80.0, CH	H-9, OH-8	C-9, -10
9	3.84 dd (8.8, 6.8)	81.5, CH	H-8, H-10	C-2, -8, -11
10	3.38 dd (7.2, 6.8)	53.5, CH	H-1, H-9	C-1, -2, -8, -9, -11, -12, -14, -17
11		147.8, C		
12	2.28 m; 2.03 m	31.5, CH_2_	H_2_-13	n.o.
13	1.69 m; 1.10 m	25.4, CH_2_	H_2_-12, H-14	n.o.
14	1.48 m	39.0, CH	H-1, H_2_-13, H-18	C-18
15	1.38 s	22.9, CH_3_		C-2, -3, -4
16	1.29 s	18.4, CH_3_		C-6, -7, -8
17	4.91 br s	111.1, CH_2_		C-10, -11, -12
	4.79 dd (2.0, 1.6)			
18	1.92 m	34.0, CH	H-14, H_2_-19, H_3_-20	C-19
19	3.95 d (6.4)	67.5, CH_2_	H-18	C-14, -18, -20, acetate carbonyl
20	0.84 d (7.2)	10.7, CH_3_	H-18	C-14, -18, -19
3-*n*-butyrate		172.3, C		
	2.32 m	37.3, CH_2_	H_2_-3′	C-1′, -3′, -4′
	1.66 m	18.4, CH_2_	H_2_-2′, H_3_-4′	C-1′, -2′, -4′
	0.99 t (7.2)	13.7, CH_3_	H_2_-3′	C-2′, -3′
6-OAc		171.9, C		
	2.08 s	21.4, CH_3_		Acetate carbonyl
19-OAc		171.2, C		
	2.09 s	21.1, CH_3_		Acetate carbonyl
OH-7	2.36 s			C-6, -7, -16
OH-8	1.82 d (9.6)		H-8	C-7, -8

*^a^* n.o. = not observed.

**Figure 1:**
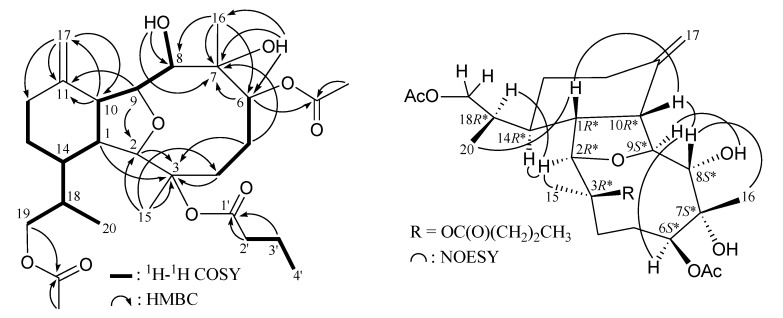
Selective key ^1^H–^1^H COSY, HMBC and NOESY correlations for **1**.

^1^H–^1^H couplings in the COSY spectrum of **1** enabled identification of the C-4/-5/-6, C-8/-9/-10/-1/-14/-13/-12, C-14/-18/-19 and C-18/-20 units (Table 1 and Figure 1), which were assembled with the assistance of an HMBC experiment. The HMBC correlations between protons and quaternary carbons of **1** (Table 1 and Figure 1), such as H-1, H-2, H_2_-4, H_2_-5/C-3; H_2_-5, H-6/C-7; and H-9, H-10, H_2_-17/C-11, permitted the elucidation of the main carbon skeleton of **1**. The exocyclic carbon-carbon double bond at C-11 was confirmed by the HMBC correlations between H-10/C-17 and H_2_-17/C-10, -11, -12. The ether bridge between C-2 and C-9 was supported by an HMBC correlation between H-9/C-2. The C-15 and C-16 tertiary methyls bonded to the C-3 and C-7 oxygenated quaternary carbons were established by the HMBC correlations between H_3_-15/C-2, -3, -4 and H_3_-16/C-6, -7, -8, respectively. The hydroxy proton signal at δ_H_ 1.82 was revealed by its ^1^H–^1^H COSY and HMBC correlations to δ_H_ 3.58 (H-8) and δ_C_ 80.0 (CH-8), respectively, indicating its attachment to C-8. The location of a hydroxy group at C-7, an oxygenated quaternary carbon, was confirmed by the HMBC correlations between a hydroxy proton at δ_H_ 2.36 and C-6, -7 and C-16. Furthermore, the acetoxy groups at C-6 and C-19 were confirmed by the HMBC correlations from oxymethine (δ_H_ 5.72, H-6) and acetate methyl (δ_H_ 2.08) to the ester carbonyl at δ_C_ 171.9 (C); and oxymethylene (δ_H_ 3.95, H_2_-19) and acetate methyl (δ_H_ 2.09) to the ester carbonyl at δ_C_ 171.2 (C), respectively. Thus, the remaining *n*-butyrate ester had to be positioned at C-3, an oxygen-bearing quaternary carbon resonating at δ_C_ 86.0 ppm. Based on the above findings, the planar structure of **1** was established.

Naturally occurring eunicellin analogues from soft corals belonging to the genus *Cladiella* have H-1 and H-10 in the β-orientation [4]. In the NOESY experiment (Figure 1), observation of the correlations between H-10 with H-1 and H-8, suggested that H-1, H-8 and H-10 are β-oriented. Also, correlations of H-2 with H_3_-15 and H-14; H-9 with H-6 and OH-8; and H-8 with H_3_-16, suggested that H-2, H-6, H-9, H-14, Me-15 and both the hydroxy groups at C-7 and C-8 are α-oriented. The C-18 asymmetric center was assigned to be R*-configured on the basis of correlations between the β-oriented H-1 and H_3_-20 and between the α-oriented H-2 and H-18. Based on the above findings, the structure of **1** was elucidated and the chiral carbons for **1** were assigned as 1R*, 2R*, 3R*, 6S*, 7S*, 8S*, 9S*, 10R*, 14R* and 18R*. The NMR data of **1** was found to be similar to those of a known compound, krempfielin C (**6**) [5] (Chart 1). Comparison of the NMR data of them revealed that the only difference between both compounds arises from the replacement of the C-19 methyl at C-18 in **6** by a acetoxymethyl group in **1**.

**Figure 2:**
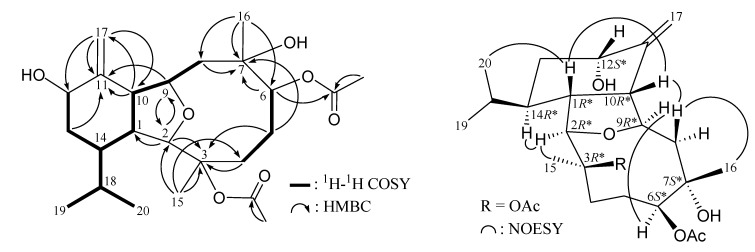
Selective key ^1^H–^1^H COSY, HMBC and NOESY correlations for **2**.

The new metabolite cladieunicellin N (**2**) was found to have the molecular formula C_24_H_38_O_7_ and six degrees of unsaturation, as indicated from the HRESIMS at *m/z* 461.25067 (calcd for C_24_H_38_O_7_Na, 461.25097). NMR data of **2** (Table 2 and Table 3) showed the presence of two acetoxy group (δ_H_ 2.08 and 2.06, each 3H × s; δ_C_ 169.5 and 22.4; 171.8 and 21.4). The ^1^H and ^13^C NMR data of **2** was found to be similar to those of a known compound, krempfielin L (**7**) (Chart 1) [6]. By comparison of the 1D and 2D NMR data of these two compounds revealed that the hydroxy group at C-6 in **7** was replaced by an acetoxy group in **2** (Table 2 and Table 3; Figure 2). The stereochemistry of **2** was confirmed by comparison of the NMR data and NOESY correlations of eunicellins **7** and **2** (Table 2 and Table 3; Figure 2).

**Table 2:** ^1^H NMR data for eunicellins **2**–**5**.

	2	3	4	5
	δ_H_ *^a^*	δ_H_ *^a^*	δ_H_ *^a^*	δ_H_ *^a^*
1	2.22 dd (10.0, 7.2) *^b^*	2.24 dd (11.2, 7.6)	2.23 dd (11.6, 6.8)	2.23 dd (10.8, 7.2)
2	3.71 s	3.71 s	3.67 s	3.62 s
4	2.55 dd (14.8, 8.8)	2.58 dd (14.8, 8.4)	2.52 dd (14.8, 8.4)	2.54 dd (14.8, 8.4)
	2.03 m	2.01 m	2.00 m	1.98 m
5	1.52 m	1.56 m	1.53 m	1.52 m
	1.45 m	1.46 dd (10.0, 6.0)	1.43 dd (9.6, 6.8)	1.47 dd (9.2, 6.4)
6	5.61 d (5.6)	5.63 d (6.0)	5.64 d (5.6)	5.84 dd (6.0, 1.2)
8	1.88 m; 1.82 m	1.88 m; 1.82 m	3.43 dd (10.8, 9.2)	3.55 dd (9.2, 9.2)
9	4.49 ddd (7.2, 6.4, 6.4)	4.37 ddd (10.0, 7.2, 5.2)	3.95 dd (9.2, 6.8)	3.83 dd (9.2, 6.8)
10	2.94 dd (7.2, 7.2)	3.00 dd (7.6, 7.2)	3.34 dd (6.8, 6.8)	3.31 dd (7.2, 6.8)
12	4.40 dd (4.0, 2.4)	5.48 dd (4.0, 2.8)	5.41 dd (4.0, 2.8)	2.29 ddd (14.0, 3.6, 3.6)
				2.05 m
13	1.89 m	1.93 ddd (14.0, 4.0, 4.0)	1.90 ddd (14.0, 4.0, 4.0)	1.75 m
	1.30 dd (12.8, 11.6)	1.30 ddd (14.0, 14.0, 2.8)	1.31 m	1.06 m
14	1.86 m	1.71 m	1.64 m	1.27 m
15	1.41 s	1.42 s	1.38 s	1.38 s
16	1.19 s	1.20 s	1.25 s	1.28 s
17	5.00 d (1.2)	5.14 d (1.6)	5.22 d (2.0)	4.87 br s
	4.81 d (1.2)	4.93 br s	5.17 d (2.0)	4.77 br s
18	1.80 m	1.83 m	1.77 m	1.73 m
19	0.98 d (6.4)	0.95 d (6.8)	0.93 d (7.2)	0.96 d (6.8)
20	0.80 d (6.8)	0.79 d (7.2)	0.77 d (6.4)	0.78 d (6.8)
3-*n*-butyrate			2.29 t (6.8)	
			1.62 sext (6.8)	
			0.94 t (6.8)	
3-OAc	2.08 s	2.09 s		2.10 s
6-OAc	2.06 s	2.07 s	2.06 s	2.07 s
12-OAc		2.04 s	2.03 s	
6-OH				
7-OH		2.32 br s	2.57 br s	2.43 br s
8-OH			2.80 d (10.8)	1.93 d (9.2)

*^a^* ^1^H spectra recorded at 400 MHz in CDCl_3_; *^b^* *J* values (Hz) in parentheses.

**Table 3:** ^13^C NMR data for eunicellin **2**–**5**.

	2	3	4	5
	δ_C_ *^a^*	δ_C_ *^a^*	δ_C_ *^a^*	δ_C_ *^a^*
1	44.8, CH *^b^*	44.7, CH	44.3, CH	45.7, CH
2	91.2, CH	91.1, CH	91.8, CH	92.4, CH
3	86.7, C	86.7, C	86.1, C	86.3, C
4	35.2, CH_2_	35.4, CH_2_	34.9, CH_2_	35.2, CH_2_
5	29.2, CH_2_	29.1, CH_2_	28.6, CH_2_	28.5, CH_2_
6	83.9, CH	84.3, CH	81.8, CH	82.1, CH
7	75.4, C	75.4, C	78.3, C	78.2, C
8	46.2, CH_2_	46.1, CH_2_	79.6, CH	80.0, CH
9	79.9, CH	79.2, CH	82.5, CH	81.4, CH
10	51.5, CH	51.8, CH	51.1, CH	53.4, CH
11	147.8, C	142.8, C	143.2, C	148.5, C
12	71.1, CH	72.8, CH	73.6, CH	31.8, CH_2_
13	30.6, CH_2_	28.5, CH_2_	28.6, CH_2_	24.9, CH_2_
14	35.6, CH	36.4, CH	37.1, CH	44.2, CH
15	23.0, CH_3_	23.0, CH_3_	23.0, CH_3_	22.8, CH_3_
16	23.5, CH_3_	23.7, CH_3_	18.3, CH_3_	18.4, CH_3_
17	113.2, CH_2_	116.7, CH_2_	117.8, CH_2_	110.7, CH_2_
18	28.6, CH	28.5, CH	28.6, CH	29.0, CH
19	21.8, CH_3_	21.7, CH_3_	21.7, CH_3_	21.9, CH_3_
20	15.6, CH_3_	15.3, CH_3_	15.4, CH_3_	15.6, CH_3_
3-*n*-butyrate			172.3, C	
			37.2, CH_2_	
			18.3, CH_2_	
			13.6, CH_3_	
3-OAc	169.5, C	169.4, C		169.6, C
	22.4, CH_3_	22.4, CH_3_		22.4, CH_3_
6-OAc	171.8, C	171.8, C	171.8, C	171.8, C
	21.4, CH_3_	21.4, CH_3_	21.4, CH_3_	21.4, CH_3_
12-OAc		170.4, C	170.8, C	
		21.6, CH_3_	21.4, CH_3_	

*^a^* ^13^C spectra recorded at 100 MHz in CDCl_3_; *^b^* Deduced from DEPT and HMQC spectra.

**Figure 3:**
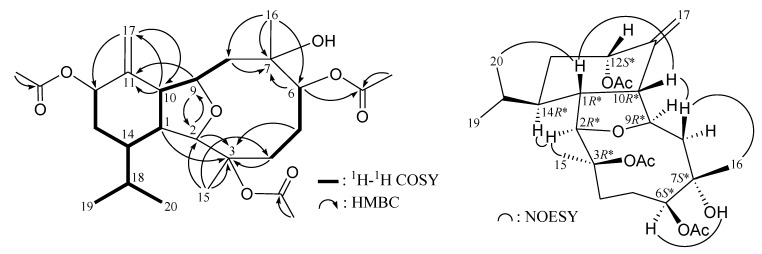
Selective key ^1^H–^1^H COSY, HMBC and NOESY correlations for **3**.

The HRESIMS of cladieunicellin O (**3**) at *m/z* 503.26152 established the molecular formula of C_26_H_40_O_8_ (calcd for C_26_H_40_O_8_Na, 503.26154). Detailed analysis shows that the NMR data of **3** (see Table 2 and Table 3) are almost identical with those of **2** except for the presence of an additional acetoxy group in **3** (δ_H_ 2.04, 3H, s; δ_C_ 170.4 and 21.6) in **3**. Furthermore, the placement of an acetoxy group at C-12 was established by the HMBC experiment which showed correlations from an oxymethine proton (δ_H_ 5.48) and acetate methyl (δ_H_ 2.04) to the ester carbonyl at δ_C_ 170.4 (C) (Figure 3). The NOESY correlations of **3** (Figure 3) also showed that the relative stereochemistry of this metabolite is similar with that of **2**. Thus the structure of eunicellin **3** was elucidated.

**Figure 4:**
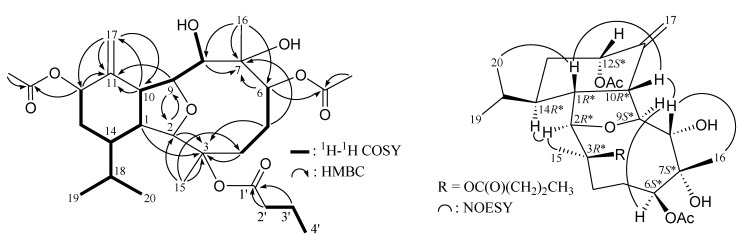
Selective key ^1^H–^1^H COSY, HMBC and NOESY correlations for **4**.

Cladieunicellin P (**4**) had the same molecular formula as that of **1**, C_28_H_44_O_9_, as determined by HRESIMS, with seven degrees of unsaturation. In the HMBC spectrum, the ^13^C signal at δ_C_ 172.3 correlated with the signal of the methylene protons at δ_H_ 2.29 (Figure 4) and was consequently assigned as the carbon atom of the *n*-butyrate carbonyl. The positions of the two acetoxy groups at C-6 and C-12, were confirmed by the correlations the two methine protons at δ_H_ 5.64 (H-6) and 5.41 (H-12) and the ester carbonyls at δ_C_ 171.8 (s) and 170.8 (s), respectively, in the HMBC spectrum of **4**. Thus, the remaining *n*-butyrate group was at C-3, an oxygenated quaternary carbon which bonded to the C-15 tertiary methyl and was confirmed by the HMBC correlations between H_3_-15/C-2, -3, -4. The relative configuration of **4** was mostly confirmed to be the same as that of **1** by comparison of the chemical shifts of both compounds (Table 1, Table 2 and Table 3) and was further confirmed by NOESY correlations (Figure 4). The coupling constants between H-12 and C-13 methylene protons (*J* = 4.0, 2.8 Hz) indicated that H-12 was positioned on equatorial direction and possessed a β-orientation in the cyclohexane ring of **4**.

**Figure 5:**
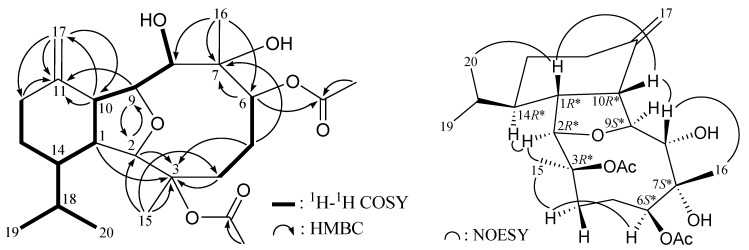
Selective key ^1^H–^1^H COSY, HMBC and NOESY correlations for **5**.

Cladieunicellin Q (**5**) exhibited the molecular ion peak [M + Na]^+^ at *m/z* 461.25110 in the HRESIMS and established a molecular formula of C_24_H_38_O_7_ (calcd for C_24_H_38_O_7_Na, 461.25097), appropriate with six degrees of unsaturation. The IR absorptions at υ_max_ 3462 and 1732 cm^−1^ revealed the presence of hydroxy and ester carbonyl functionalities. The ^13^C NMR spectrum of **5** showed signals of 24 carbons (Table 3), which were characterized by the DEPT spectrum of six methyls (including two acetate methyls), five methylenes (including an sp^2^ methylene), eight methines (including four oxymethines) and five quaternary carbons (including two ester carbonyls and an sp^2^ quaternary carbon of an olefin). The ^1^H and ^13^C NMR spectral data of **5** (Table 2 and Table 3) also showed the presence of two acetoxy groups (δ_H_ 2.10 and 2.07, each 3H × s; δ_C_ 22.4 and 21.4, acetate methyls; δ_C_ 169.6 and 171.8, acetate carbonyls). The remaining three degrees of unsaturation identified **5** as a tricyclic diterpenoid. The molecular framework was established by ^1^H–^1^H COSY and HMBC correlations (Figure 5). Comparison of the NMR data of **5** with those of the known compound, cladieunicellin L (**8**) [2] revealed that **5** is the 12-deacetoxy derivative of cladieunicellin L. The stereochemistry of compound **5** was determined by the NOESY spectrum as shown in Figure 5.

Cytotoxicity of compounds **1**–**5** toward Molt 4 (human acute lymphoblastic leukemia) and HL 60 (human promyelocytic leukemia) cells was studied, and the results are shown in Table 4. Eunicellins **1**, **3** and **5** was found to exhibit moderate cytotoxicity against Molt 4 cells. Eunicellin **2** did not show cytotoxicity toward Molt 4 cells, implying that the presence of a hydroxy substituent at C-12 would weaken the activity comparison with the structure and cytotoxicity of **3**. Eunicellin **4** was found to be inactive against Molt 4 cells, indicating that the bulky *n*-butyrate group at C-3 could reduce cytotoxicity in comparison with the structure and cytotoxicity of cladieunicellin L (**8**) [2].

**Table 4:** Cytotoxic data of compounds **1**−**5**.

	Cell Lines IC_50_ (μM)	
Compounds	Molt 4	HL 60
**1**	16.43	>20
**2**	>20	>20
**3**	14.17	>20
**4**	>20	>20
**5**	15.55	>20
**8 ** *^a^*	14.42	>20
Doxorubicin *^b^*	0.02	0.02

*^a^* Data was reported in [2]; *^b^* Doxorubicin was used as a positive control.

**Chart 2:**
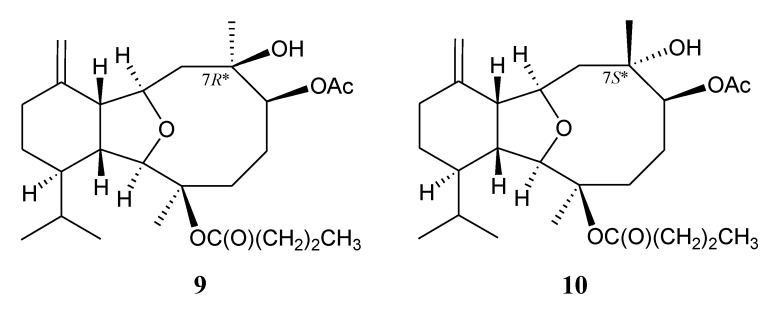
The structures of litophynin I diacetate (**9**) and its revised structure **10**.

In a previous study, we reported the isolation of a natural eunicellin, litophynin I diacetate (**9**) [1,7]. However, based on the spectral data analysis and by comparing the ^13^C NMR chemical shifts of C-7 and C-16 with those of its analogues [8], the C-7 should be revised as to possess an S*-configuration as presented in eunicellin **10** (Chart 2).

## 3. Experimental Section

### 3.1. General Experimental Procedures

Optical rotations were measured on a Jasco P-1010 digital polarimeter (Japan Spectroscopic Corporation, Tokyo, Japan). Infrared spectra were recorded on a Varian Diglab FTS 1000 FT-IR spectrometer (Varian Inc., Palo Alto, CA, USA) or a Jasco 4100 FT-IR spectrometer (Japan Spectroscopic Corporation, Tokyo, Japan); peaks are reported in cm^−1^. NMR spectra were recorded on a Varian Mercury Plus 400 NMR spectrometer (Varian Inc., Palo Alto, CA, USA) using the residual CHCl_3_ signal (δ_H_ 7.26 ppm) as the internal standard for ^1^H NMR and CDCl_3_ (δ_C_ 77.1 ppm) for ^13^C NMR. Coupling constants (*J*) are given in Hz. ESIMS and HRESIMS were recorded using a Bruker 7 Tesla solariX FTMS system (Bruker, Bremen, Germany). Column chromatography was performed on silica gel (230–400 mesh, Merck, Darmstadt, Germany). TLC was carried out on precoated Kieselgel 60 F_254_ (0.25 mm, Merck, Darmstadt, Germany); spots were visualized by spraying with 10% H_2_SO_4_ solution followed by heating. The normal phase HPLC (NP-HPLC) was performed using a system comprised of a Hitachi L-7110 pump (Hitachi Ltd., Tokyo, Japan) and a Rheodyne 7725 injection port (Rheodyne LLC, Rohnert Park, CA, USA). Two normal phase columns (Supelco Ascentis^®^ Si Cat #:581515-U, 25 cm × 21.2 mm, 5 μm; 581514-U, 25 cm × 10 mm, 5 μm, Sigma-Aldrich, St. Louis, MO, USA) were used for NP-HPLC.

### 3.2. Animal Material

Specimens of the octocoral *Cladiella* sp. [9] were collected by hand using SCUBA equipment off the coast of Penghu Archipelago, Taiwan on September 2011, and stored at −20 °C until extraction. A voucher specimen (NMMBA-TWSC-11011) was deposited in the National Museum of Marine Biology and Aquarium, Taiwan.

### 3.3. Extraction and Isolation

Specimens of the soft coral *Cladiella* sp. (wet weight 1.25 kg, dry weight 457 g) were minced and extracted with ethyl acetate (EtOAc). The EtOAc extract left after removal of the solvent (12.4 g) was separated by silica gel and eluted using *n*-hexane/EtOAc in a stepwise fashion from 100:1 to pure EtOAc to yield 17 fractions A–Q. Fraction O (716 mg) was chromatographed on silica gel, using a mixture of *n*-hexane and acetone in a stepwise fashion from 6:1 to pure acetone to obtain 12 subfractions O1–O12. Fractions O4 (57.0 mg) and O5 (258.9 mg) were repurified by NP-HPLC, using a mixture of dichloromethane and acetone to yield **3** (8:1, flow rate: 3.0 mL/min, 7.1 mg, *t*_R_ = 91 m) and **5** (8:1, flow rate: 3.0 mL/min, 15.0 mg, *t*_R_ = 66 m), respectively. Fraction O6 (170.7 mg) was repurified by NP-HPLC, using a mixture of dichloromethane and acetone (7:1, flow rate: 3.0 mL/min) to yield **1** (4.8 mg, *t*_R_ = 80 m) and **4** (69.9 mg, *t*_R_ = 96 m), respectively. Fraction Q (930 mg) was separated by silica gel, using a mixture of *n*-hexane and acetone in a stepwise fashion from 3:1 to pure acetone to obtain 15 subfractions Q1–Q15. Fraction Q3 was repurified by NP-HPLC, using a mixture of *n*-hexane and acetone (2:1, flow rate: 3 mL/min) to yield **2** (15.6 mg, *t*_R_ = 76 min).

Cladieunicellin M (**1**): Colorless oil; 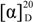 −10 (*c* 0.1, CHCl_3_); IR (neat) υ_max_ 3462, 1734 cm^−1^; ^1^H (400 MHz, CDCl_3_) and ^13^C (100 MHz, CDCl_3_) NMR data, see Table 1; ESIMS: *m/z* 547 [M + Na]^+^; HRESIMS: *m/z* 547.28760 (calcd for C_2__8_H_44_O_9_Na, 547.28775).

Cladieunicellin N (**2**): Colorless oil; 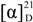 +31 (*c* 0.8, CHCl_3_); IR (neat) υ_max_ 3437, 1729 cm^−1^; ^1^H (400 MHz, CDCl_3_) and ^13^C (100 MHz, CDCl_3_) NMR data, see Table 2 and ; ESIMS: *m/z* 461 [M + Na]^+^; HRESIMS: *m/z* 461.25067 (calcd for C_24_H_38_O_7_Na, 461.25097).

Cladieunicellin O (**3**): Colorless oil; 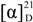 +14 (*c* 0.4, CHCl_3_); IR (neat) υ_max_ 3478, 1729 cm^−1^; ^1^H (400 MHz, CDCl_3_) and ^13^C (100 MHz, CDCl_3_) NMR data, see Table 2 and ; ESIMS: *m/z* 503 [M + Na]^+^; HRESIMS: *m/z* 503.26152 (calcd for C_26_H_40_O_8_Na, 503.26154).

Cladieunicellin P (**4**): Colorless oil; 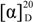 −7 (*c* 3.0, CHCl_3_); IR (neat) υ_max_ 3448, 1733 cm^−1^; ^1^H (400 MHz, CDCl_3_) and ^13^C (100 MHz, CDCl_3_) NMR data, see Table 2 and ; ESIMS: *m/z* 547 [M + Na]^+^; HRESIMS: *m/z* 547.28755 (calcd for C_28_H_44_O_9_Na, 547.28775).

Cladieunicellin Q (**5**): Colorless oil; 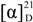 +24 (*c* 0.6, CHCl_3_); IR (neat) υ_max_ 3462, 1732 cm^−1^; ^1^H (400 MHz, CDCl_3_) and ^13^C (100 MHz, CDCl_3_) NMR data, see Table 2 and ; ESIMS: *m/z* 461 [M + Na]^+^; HRESIMS: *m/z* 461.25110 (calcd for C_24_H_38_O_7_Na, 461.25097).

### 3.4. MTT Antiproliferative Assay

HL 60 (human promyelocytic leukemia) and Molt 4 (Human acute lymphoblastic leukemia) cells were obtained from the American Type Culture Collection (ATCC, Manassas, VA, USA). Cells were maintained in RPMI 1640 medium supplemented with 10% fetal calf serum, 2 mM glutamine, and antibiotics (100 units/mL penicillin and 100 μg/mL streptomycin) at 37 °C in a humidified atmosphere of 5% CO_2_. Cells were seeded at 4 × 10^4^ per well in 96-well culture plates before treatment with different concentrations of the tested compounds. The compounds were dissolved in dimethyl sulfoxide (less than 0.02%) and made immediately of 1.25, 2.5, 5, 10 and 20 μg/μL prior to experiments. After treatment for 72 h, the cytotoxicity of the tested compounds was determined using MTT cell proliferation assay (thiazolyl blue tetrazolium bromide, Sigma-M2128, St. Louis, MO, USA). The MTT is reduced by the mitochondrial dehydrogenases of viable cells to a purple formazan product. The MTT-formazan product dissolved in DMSO. Light absorbance values (OD = OD_570_ − OD_620_) were recorded at wavelengths of 570 and 620 nm using an ELISA reader (Anthos labtec Instrument, Salzburg, Austria) for calculating the concentration which caused 50% inhibition (IC_50_), *i.e.*, the cell concentration at which the light absorbance value of the experimental group is half that of the control group. These results were expressed as a percentage of the control ± SD established from *n* = 4 wells per one experiment from three separate experiments [10].

## 4. Conclusions

Five new 7α-hydroxyeunicellin-based diterpenoids, cladieunicellins M–Q (**1**–**5**), were isolated from the soft coral *Cladiella* sp. The eunicellins **1**, **3** and **5** are found to show moderate cytotoxicity against the Molt 4 human acute lymphoblastic leukemia. The soft coral *Cladiella* sp. will be transplanted to culturing tanks located in the National Museum of Marine Biology and Aquarium, Taiwan, for extraction of additional natural products to establish a stable supply of bioactive material.
